# Immune cells as a potential therapeutic agent in the treatment of depression

**DOI:** 10.1192/j.eurpsy.2022.694

**Published:** 2022-09-01

**Authors:** E. Markova, M. Knyazheva

**Affiliations:** Federal State Research Institute of Fundamental and Clinical Immunology, Neuroimmunology Lab., Novosibirsk, Russian Federation

**Keywords:** immune cells, Depression

## Abstract

**Introduction:**

There are sufficient amount data on the immune cells and their biologically active products leading role in the pathogenesis of depression, which allows viewing modulated immune cells as model objects for developing new approaches to depression immunotherapy.

**Objectives:**

We first demonstrated the ability of immune cells modulated outside the body by caffeine to edit depression-like behavior and showed the central cytokines-mediated mechanisms of this effect. Considering theimportant role of the peripheral immune system in the pathogenesis of depression, we investigated the main parameters of its functional activity after transplantation of modulated immunocytes.

**Methods:**

(CBAxC57Bl/6) F1 depressive-like male mice, developed under the long-term social stress, were undergoing the transplantation of syngeneic immune cells with *in vitro* caffeine-modulated functional activity. Recipient’s behavior and immune systems functional activity parameters were studied.

**Results:**

We showed earlier significant positive psychoneuromodulatory effect of caffeine-treated immune cells in depressive-like recipients which manifests itself in the behavioral editing (anhedonia reduction, stimulation of exploratory behavior and activity in forced swimming test); hippocampal neurogenesis stimulation against the background of increased BDN and modulation of brain cell’s cytokines production. Transplantation of caffeine-modulated immune cells in syngeneic depressive-like recipients also leads to positive changes in the immune system functional activity as evidenced by enhanced immune response, splenocytes proliferation stimulation on the background of modulation of cell’s cytokines production and decreased tryptophan catabolism, reducing systemic inflammation.

**Conclusions:**

Results demonstrated that *in vitro* caffeine-modulated immune cells caused positive psychoneuroimmunomodulating effect in depressive-like recipients. So, its may be considered as a potential therapeutic agent in the treatment of depression.

**Disclosure:**

No significant relationships.

